# Facilitators and barriers for the delivery and uptake of cervical cancer screening in Indonesia: a scoping review

**DOI:** 10.1080/16549716.2021.1979280

**Published:** 2021-09-29

**Authors:** Gianna Maxi Leila Robbers, Linda Rae Bennett, Belinda Rina Marie Spagnoletti, Siswanto Agus Wilopo

**Affiliations:** a Nossal Institute of Global Health, The University of Melbourne, Melbourne, Australia; b Center for Reproductive Health, Faculty of Medicine, Public Health and Nursing, Universitas Gadjah Mada, Yogyakarta, Indonesia

**Keywords:** Gynaecological cancer, female cancer, secondary prevention, sexual and reproductive health and rights, visual inspection with acetic acid

## Abstract

**Background:**

Cervical cancer (CC) is the second most common female cancer. In Indonesia, national CC screening coverage is low at 12%, highlighting the need to investigate facilitators and barriers to screening.

**Objective:**

This review synthesises research on facilitators and barriers to the delivery and uptake of CC screening; analyses them in terms of supply- and demand-side factors and their interconnectedness; and proposes recommendations for further research.

**Methods:**

Medline Ovid, CINAHL, Global Health, Neliti, SINTA and Google Scholar were searched, applying a search string with keywords relevant to screening, CC and Indonesia. In total 34 records were included, all were publications on CC screening in Indonesia (2000-2020) in English or Indonesian. Records were analysed to identify findings relevant to the categories of barriers and facilitators, supply-and demand-side factors.

**Results:**

Demand-side facilitators identified included:  husband, family or social/peer support (14 studies); information availability, knowledge and awareness (12 studies); positive attitudes and strong perception of screening benefit and the seriousness of CC (12 studies); higher education and socioeconomic status (11 studies); having health insurance; and short distance to screening services (4 studies). Evidence on supply-side was limited. Supply-side facilitators included counselling and support (6 studies), and ease of access (6 studies). Demand-side barriers identified focused on: lack of knowledge/awareness and lack of confidence in screening (14 studies); fear, fatalism and shame (10 studies); time and transportation constraints (8 studies); and lack of husband approval and support (6 studies). Supply-side barriers included: lack of skilled screening providers (3 studies); lack of advocacy and health promotion (3 studies); resource constraints (3 studies); and lack of supervision and support for health care providers (3 studies).

**Conclusions:**

Facilitators and barriers were mirrored in the supply- and demand-side findings. The geographical scope and population diversity of existing research is limited and further supply-side research is urgently needed.

## Background

Cervical cancer (CC) is the fourth most common cancer in women worldwide and one of the most common cancers in low- and middle-income countries (LMICs) [1]. In 2018, 84% of all new CC diagnoses as well as 90% of CC-related deaths affected women in LMICs [2]. Reproductive cancers contribute to the rising burden of chronic diseases worldwide, which disproportionally affect LMICS in particular Southeast Asia [3,4]. In addition, three times more women die from reproductive cancers than women die from childbirth complications every year [5]. In Indonesia, chronic diseases including cancer dominate the country’s mortality rates and contribute to 73% of all deaths [6,7]. Women in LMICs die disproportionally from reproductive cancers compared to women in high-income countries due to lack of access to cost-effective and life-saving interventions [5]. Many LMICs also struggle with inadequate and fragmented health systems ill-equipped to attend to the screening needs of all women, further reinforcing the disadvantage and cycle of poverty already experienced by vulnerable women [5].

**Table 1.** Description of included articles

Characteristics of research	n*	References
Quantitative methodology	27	[23,34–38,41–43,45–50,52–63]
Qualitative methodology	6	[22,33,39,40,44,51]
Mixed- methodology	1	[31]
Primary Data	29	[22,33–44,46,47,50–63]
Secondary Data	3	[23,49,63]
Primary & Secondary Data	3	[31,45,48]
Population Sample		
Women only	29	[23,33–38,41–43,45–63]
Health Care Providers**	4	[22,31,39,44]
Men**	3	[22,31,40]
Location		
Rural	6	[35–40]
Peri-urban	2	[33,34]
Urban	23	[22,31,43–63]
Urban & Rural	2	[41,42]
National (13/27 provinces)	1	[23]
Provinces		
Central Java	13	[35–38,40,42,44,45,52,53,56,61,62]
East Java	5	[33,46,49,54,55]
West Java	4	[22,47,50,57]
Jakarta	3	[31,51,63]
Yogyakarta	3	[34,41,43]
North Sumatra	1	[59]
Riau	1	[39]
South Sulawesi	1	[48]
Banten	1	[58]
Bali	1	[60]
Timeframe of Publication		
2000–2010	1	[31]
2015–2020	29	[22,23,33–55,57,59–63]

**Studies counted more than once per category

*Number of studies

**Table 2.** Identified themes

Theme	n*	References
Demand-Side Facilitators:		
Husband, Family or Social/Peer Support	14	[22,23,35–38,40,45,49,53,55–57,62]
Information availability, knowledge and awareness	12	[34,36,40,45,46,48,49,51–53,55–58,61,62]
Positive attitude, motivation and perception	12	[37,38,43,49,51–54,58,60–62]
Having health insurance and short travel insurance	4	[23,41,42,45]
Higher education and socioeconomic status	11	[23,38,47,50,52,55,57,58,61–63]
Supply-Side Facilitators:		
Counselling and Support	6	[22,33,37–39,47]
Ease of access	6	[22,33,41,42,51,53]
Health Promotion and Advocacy	2	[22,39]
HCPs gender	2	[40,41]
Clear supervision, support and ensuring quality of services	1	[44]
Demand-Side Barriers:		
Lack of knowledge, awareness and lack of confidence	14	[22,23,31,33,34,36,40,48,51,55,57–59,62]
Low-risk perception	3	[40,50,51,55]
Lack of husband’s permission and general support	6	[22,31,41,47,50,56,59]
Fear, fatalism and shame	10	[22,33,34,39–41,47,50,51,55]
Time and transport constraints	8	[22,31,41,42,46,50–52,57]
Low education	4	[45,58,59,63]
Supply-Side Barriers:		
Limited access/coverage and operating hours	2	[22,31]
Lack of skilled CC screening providers	3	[36,39,44]
Lack of advocacy and health promotion	3	[22,44,52]
Inadequate implementation & coordination	2	[40, 45]
Resource constraints	3	[23, 40]
Lack of communication and support for HCPs	3	[23, 40, 45]

A data analysis of 185 countries from the Global Cancer Observatory (Globocan) 2018 database, showed that Africa accounts for the highest CC incidence and mortality rates worldwide due to high rates of HIV, followed by South-eastern Asia [1,8]. Within Asia, Indonesia accounts for one of the highest CC age-standardised incidence (approx. 24 per 100,000 women-years) and mortality rates (approx. 15 per 100,000 women-years) [1]. Moreover, Indonesia’s reported CC incidence doubled between 2012 and 2018 [9]. This jump may reflect the Government of Indonesia’s (GoI) introduction of universal health coverage (UHC) through the National Health Insurance Scheme (JKN) in 2014, which resulted in CC treatment becoming free of charge and subsequently more women are presenting for diagnosis and treatment [10]. The introduction of UHC indicates the GoI’s commitment towards realising its citizens’ right to health. However, women’s fulfilment of their right to comprehensive sexual and reproductive health care in relation to the prevention and early detection of CC remains insufficient. At present, 70% of Indonesian women are diagnosed at advanced stages of CC and 50% of all Indonesian women diagnosed die from the disease [11,12]. Fifty Indonesian women are now dying daily from CC [13]. As a result of the significant burden of CC, Indonesia has signed the World Health Organization (WHO)’s Global Strategy to Accelerate the Elimination of CC launched in 2020. The GoI has committed to screen at least 70% of women between the ages 35 and 45 and to enable 90% of women diagnosed with CC to receive treatment by 2030 [14,15]. While our focus in this review is on Indonesia, the review contributes to the larger global project of developing and interpreting a sufficient evidence-base to tackle the vast inequity in access to life-saving cervical cancer screening among women living in LMIC.

## Cervical cancer screening in Indonesia

CC prevention includes primary and secondary prevention and should engage women across their life-course. For women who are sexually active,^1^^1^Since 2015, there also have been efforts to implement a school-based HPV vaccination program for adolescent girls before sexual debut. However, due to logistical issues in vaccine availability and administration changes within the Ministry of Health in late 2019, the program has been temporarily stalled [11,78]. the World Health Organization (WHO) recommends a screen-and-treat program that prioritises women aged 30–49, and repeat screening every 3–5 years [16]. For lower-resource settings such as Indonesia, the visual inspection with acetic acid (VIA) screening method is recommended. Indonesia adopted the WHO-recommended model and introduced the Cervical and Breast Cancer Prevention Project, which was first piloted in Karawang district, West Java in 2007 [17]. From 2014 the full cost of certain CC screening services was covered by the National Health Insurance Scheme. Indonesia’s national CC screening program provides free services to married women aged 30–50 years, including VIA or cytology [18]. CC screening is performed every 3–5 years. For women who are screened positive for precancerous lesions, repeat exams are recommended yearly [19–21].

For low-income women, CC screening is available for free at primary health centres (*puskesmas*) or during outreach mass screening programs conducted within low-income communities [22]. While reliable data on health care coverage in Indonesia is scarce, some estimates from 2014 indicated that government-run screening programs were only available in eight out of 34 provinces [21,23,24]. The CC screening coverage reached only 12% of women in the target population (30–50 years) in 2020 [25]. There is also great variance between provinces with the lowest CC screening coverage reported in Papua (0,9%), while the highest coverage was in Bangka Belitung (25%) [25,26]. This indicates widespread inequality in access to the government-run CC screening covered by the National Insurance Scheme and significant shortfalls in capacity in public screening service delivery.

Indonesia faces manifold challenges in CC screening program implementation and uptake and a detailed assessment of existing literature is needed to inform improvements in CC screening program delivery and uptake by 2030. No comprehensive analysis of prior research on the range of barriers and facilitators influencing both the delivery and uptake of CC screening has been undertaken. This is a crucial gap, as improvements in screening coverage cannot be realised without a comprehensive understanding of the underlying dynamics and challenges from both the demand and supply sides. This scoping review analyses existing research on CC screening in Indonesia to synthesise what is currently known about factors that impede and facilitate uptake of CC screening, and to identify the strengths and weaknesses of current research findings and gaps in knowledge

A scoping review approach has been applied in this article to investigate the extent of heterogenous knowledge on the topic, to identify knowledge gaps, and to guide future research [27]. For the purpose of this scoping review we define the demand-side of the supply-demand nexus as including people requiring access to or influencing access to CC screening services. The demand-side includes women who are potential or actual consumers of such services, and their partners and family members who may influence their engagement with screening services. Supply-side factors refer to components of the health system necessary for the delivery of CC screening. While consumers of health care are increasingly considered an integral part of the health system, this review separates the supply- and demand-sides because this delineation is apparent in the literature reviewed. The supply-side components identified as influencing facilitators and barriers to screening mainly refer to: health service delivery; health service coverage; health workforce capabilities; and capacity to provide quality CC screening. Barriers to screening refer to obstacles, including beliefs and attitudes, that impede women from accessing CC screening. Barriers to screening relate to obstacles to both initial and repeat screening. Facilitators of CC screening relate to enabling conditions or actions that support and encourage women to be screened or to participate in screening. Facilitators typically create conditions which are responsive both to the individual, and to the social, cultural, geographic and economic contexts of women’s lives. Facilitators support women to engage in initial screening or repeat screening, and also support women to follow the recommended treatment if screening results are positive for pre-cancer. However, this scoping review focuses solely on screening and does not extend to a discussion of facilitators and barriers for the provision of and access to CC primary prevention or treatment for women with pre-cancerous lesions. The objectives of this scoping review are three-fold. We first synthesise relevant research on facilitators and barriers to the delivery and uptake of CC screening in Indonesia. Second, the facilitators and barriers identified are analysed in terms of supply- and demand-side factors and their interconnectedness. Finally, we identify knowledge gaps and recommend how future research can address these gaps.

## Methods

The scoping review followed the PRISMA scoping review guidelines and included peer-reviewed research articles and grey literature as defined by Auger (1998) [28], published between 2000 and 2020, in English or Indonesian, and which reported on the national CC screening program of Indonesia [27]. The time period was chosen as the national CC screening program was trialled and introduced in the early 2000s. The following five databases were used to search for eligible studies: Medline Ovid, CINAHL, Global Health, Neliti, SINTA and Google Scholar (first 10 pages of results). The Indonesian databases SINTA and Neliti were included on advice of SA to ensure the inclusion of research published only in Indonesia.

## Inclusion criteria and screening process

Inclusion criteria for records included those that: 1) explicitly discussed barriers and/or facilitators to the delivery or uptake of CC screening in Indonesia; 2) were published in either English or Indonesian; and 3) included a description of research methodology that enabled us to determine that the research was re-producible and unbiased. A search string with relevant keywords consisting of three sub-searches was developed and included the following terms: a) screening terms (pap smear* or papanicolaou* or papanicolaou test or pap test* or visual inspection* or VIA test*), b) cervix uteri terms (cervix* or cervical or cervix uteri); c) Indonesia*. Search terms for the Indonesian database Neliti and SINTA included: *deteksi dini kanker serviks or kanker serviks or leher Rahim or deteksi dini kanker leher rahim or IVA kanker or pelayanan deteksi kanker leher rahim*. GMLR is studying Indonesian and SAW, LRB and BRMS speak and read Indonesian fluently, SAW is Indonesian, ensuring clarity in comprehension of the Indonesian language articles. All sub-searches were combined to yield the most relevant results (Supplement 1).

## Data analysis

**Figure 1.**
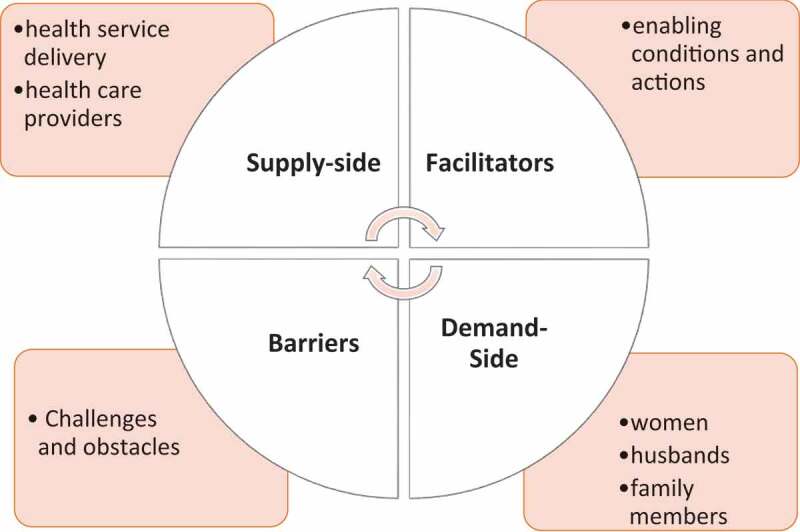
Nexus between barriers, facilitators, supply- and demand-side factors

Included literature was analysed according to the categories of barriers, facilitators, supply- and demand-side as demonstrated in Figure 1. Below, with common themes identified within each of these categories.

**Figure 2.**
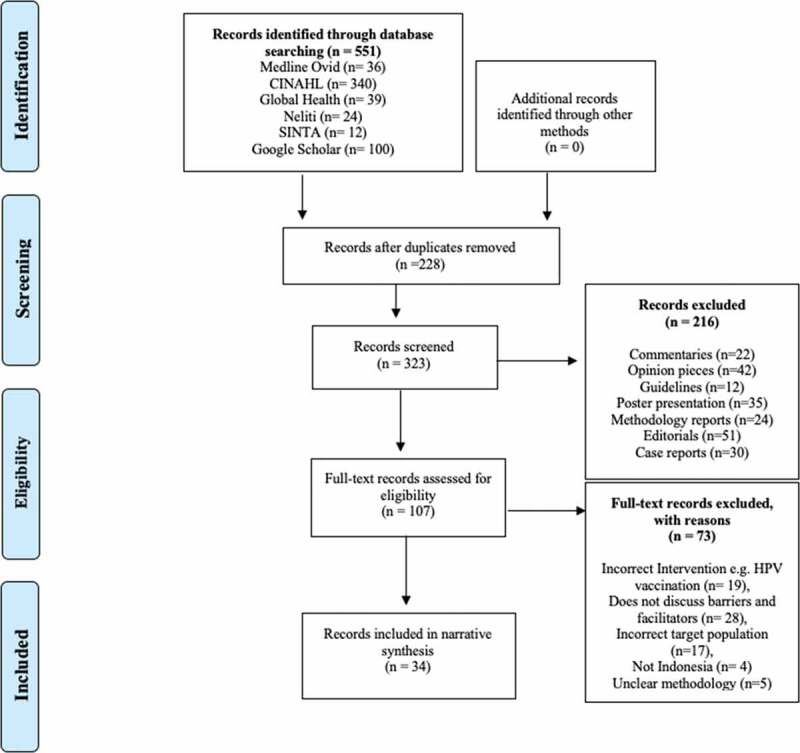
Prisma flow chart [27]

The database search initially yielded 551 records and was conducted in December 2020; 323 records remained after duplicates were removed. The 323 records were screened by GMLR for their eligibility, with Indonesian sources cross-checked by BRMS to ensure appropriate inclusion of research published in Indonesian. Evaluation and selection of records based on inclusion criteria was conducted by GMLR and BRMS in consensus. Disagreements over the inclusion of individual records were resolved via consultation with LRB. Citations that included published abstracts only, poster presentations, commentaries, opinion pieces, editorials, guidelines, methodology reports, case reports and grey literature without description of research methodology was removed. Review articles were also excluded and reference lists were scanned for any missed eligible studies. All other records were considered, provided they met the inclusion criteria. The Prisma flow chart shows the process of study selection and inclusion (Figure 2). Of 323 records, 216 were excluded after a title, keywords and abstract screening and 107 records remained for full-text screening. After full-text screening, 34 were found to be eligible for data abstraction. Citations were imported into Endnote and key data was summarised in a table (Supplement 2). The table in Supplement 2 was developed and piloted with six records by the authors. GMLR and BRMS then independently extracted and cross-checked for consistency the following data for each included record: details on author, publication date, data collection date, study type, applied research methods, study sample, research location and setting, identified facilitators and barriers on the supply- and demand-side for each included publication and recommendations given by the respective authors. The data analysis of the 34 included records included the identification of common themes among both barriers and facilitators, and analysis of how both supply- and demand-side factors influence facilitators and barriers. A deductive thematic analysis approach was applied, by identifying key themes based on pre-existing knowledge of the topic and repetitive reading of the texts to enable dominant themes to emerge across the body of research. The emerging themes of this approach are then used as categories for further analysis [29,30]. A narrative synthesis approach was then applied to describe key themes across the categories of barriers and facilitators, from both supply- and demand-side of the health system.

## Results

In total 34 records met the inclusion criteria, all of which were articles that reported on published studies from peer-reviewed journals. Table 1 provides an overview of the included literature. Out of 34 articles, 29 articles discussed studies analysing primary data (n = 29), three articles discussed studies that analysed secondary data,^2^^2^Secondary data based on the Indonesian Family Life Survey (2014–2015) [23], health data from the Ministry of Health Research and Development Agency RI collected for the cohort study of non-communicable disease risk factors 2011 [63] and medical records [49]. and three articles discussed studies that analysed both, primary and secondary data.^3^^3^Primary data was collected via questionnaires and secondary data was based on medical records/health data regularly collected by participating health centres. The included time period of secondary data was only specified by Susanti et al. (2003): year 2000–2001 [31]. Time origin of secondary data for Nordianti et al. (2018) [45] and Nuryana et al. (2019) [48] was not indicated. Of the included articles, almost two thirds were in English (n = 22); the remainder were in Indonesian (n = 12). The included articles reported on studies which primarily applied quantitative methodologies (n = 27), while six articles discussed studies that applied a qualitative methodology. One of the included articles discussed a study that applied a mixed methodology [31]. The sample sizes of the various studies ranged from 12 to 5,397 participants.

The 34 articles discussed studies that were clustered in ten of Indonesia’s 34 provinces (of which six are located on the island of Java): Central Java (n = 13), East Java (n = 5), West Java (n = 4), Jakarta (n = 3), Yogyakarta (n = 3), North Sumatra (n = 1), Riau (n = 1), South Sulawesi (n = 1), Banten (n = 1) and Bali (n = 1). One study conducted in 2018, conducted a secondary data analysis of the fifth Indonesian Family Life Survey (2014–2015) and analysed survey answers in 13 out of 27 provinces [23]. Twenty-three articles reported on studies conducted on the island of Java, indicating the historical bias toward what is known as ‘inner Indonesia’ [32]. Most articles reported studies that were conducted in urban settings (n = 23), two articles reported on studies that were conducted in peri-urban setting [33,34] and six articles reported on studies that took place in a rural setting [35–40]. Two articles discussed studies in Yogyakarta and Central Java were undertaken in both rural and urban communities [41,42]. One article analysed secondary data of a national survey, but did not disaggregate responses by area [23]. The majority of articles (n = 29) was published between 2015 and 2020. Most included articles were about studies that used health care settings to recruit participants, with 17 conducted via primary health centres (*puskesmas*) [22,34,36,39,43–55]. One article reported on a study that recruited participants via a hospital in Jakarta [31]. Four articles discussed studies that were conducted in a community setting [33,40,56,57], one in a workplace setting [58] and remaining articles reported on studies (n = 11) that did not specify the types of research sites where data was collected [23,35,37,38,41,42,59–63].

The majority of included articles (n = 25) discussed facilitators and barriers [22,23,33,34,36,39–42,44–48,50–52,54–58,60,61,63], two articles explored barriers exclusively [31,59] and seven articles focused only on facilitators [35,37,38,43,49,53,62]. Articles exploring the demand-side of CC screening predominately focused on women, with 29 articles reporting on studies having women participants only. Three articles reported on studies with both women and men as participants [22,31,40]. Four articles reported on studies that also included health care providers (HCPs), health officers or primary health clinic managers [22,31,39,44]. Research that evaluated the supply-side of CC screening mainly focused on: health worker training or skills; CC screening service coverage; health service capacity; and resource constraints leading to poor implementation and CC screening service delivery. The included research predominately focused on married women, with seven articles reporting on studies that analysed women’s CC screening uptake among married women exclusively [36,41,45,50,51,56,58]. Another five articles reported on studies that analysed answers of women with a majority of them being married, ranging between 65% and 93% of the total study sample [23,31,33,43,47]. Therefore, unmarried women’s experiences of screening are almost entirely absent or only present in small percentages of the included population sample of the individual studies.^4^^4^While some studies aggregated their population sample according to marital status incl. married, single, widowed, not all included studies provided this information.

## Facilitators of CC uptake and delivery

Two included articles discussed supply-side facilitators [39,44], 19 articles discussed demand-side facilitators [34–36,43,45,46,48–50,52,54–58,60–63], and eleven discussed demand and supply-side facilitators [22,23,33,37,38,40–42,47,51,53]. Table 2 provides an overview of the identified themes within the included literature. Five main demand-side facilitators emerged from our analysis, these are: **husband, family or social/peer support; information availability, knowledge and awareness; positive attitude, motivation and perception of benefit of screening and seriousness of CC; having health insurance and short travel distance to CC screening service**; and **women having higher education and socioeconomic status**. Ten articles discussed studies conducted across West Java, Java and East Java, Yogyakarta, Jakarta and Riau province addressed supply-side facilitators. The following four supply-side facilitator themes emerged from our analysis: **Counselling and support; ease of access; health promotion and advocacy; HCPs gender; and clear supervision and support of HCPs and ensuring quality of services.**

### Demand-side facilitators

One of the most significant demand-side facilitators identified across the literature was **husband, family or social/peer support –** referring to the support of women received from their husbands, family members, and their friends and peers. This type of support was commonly characterised as any emotional or tangible support such as active encouragement to seek CC screening, assistance to attend services or to access information or to provide an open and positive communication about the topic. Fourteen publications discussed the positive impact of such support on women’s CC screening uptake and were conducted in West, Central and East Java [22,23,35–38,40,45,49,53,55–57,62]. Husband support was stated as a significant facilitator for increasing CC screening uptake in multiple studies across West, Central and East Java (n = 6) [22,35–37,49,55]. Husband support included gaining permission from spouses to access CC screening services, being encouraged or advised by spouses to seek CC screening services and being accompanied to CC screening services by one’s husband. In a study in Central Java (n = 80), women whose husbands supported them to access CC screening were three times more likely to access CC screening services than women who did not have their husband’s permission [35]. Five articles described family support as an important facilitator to encourage women to attend CC screening [22,38,45,53,55]. Family support included the support of other relatives in terms of encouragement and tangible support to access information and services related to CC screening [22,38,45,53,55]. However, while family support was observed across multiple studies as important, husband support was established as being of critical importance in terms of influencing women’s CC screening uptake [55].

Social or peer support was discussed in eight articles and was defined as the provision of information, encouragement and support with respect to learning about CC screening and seeking services provided by friends or community members [22,23,36–38,56,57,62]. A study in Central Java (n = 100) described how advice and support of a close friend can encourage women to attend outreach screening events [36]. Another study in Central Java (n = 99) found that women who had strong social support are up to ten times more likely to attend CC screening than women without significant social or peer support [56]. An evaluation of the fifth Indonesian Family Life Survey data (2014–2015) (n = 5,397) asserted that women who regularly participated in social activities and interacted with peers and community members were more likely to be exposed to CC screening information and to access screening [23].

Twelve studies described the importance of **information availability, knowledge and awareness** of CC and screening services as facilitators of screening uptake, these studies were conducted across West, East and Central Java, South Sulawesi, Yogyakarta, Jakarta and Banten [34,36,40,45,46,48,49,51–53,55–58,61,62]. Another study tested the significance of a health education intervention on improving women’s knowledge of CC screening and the CC screening behaviour of study participants (n = 79) [46]. The intervention used audio-visual material and information booklets on CC screening, and found that women in the intervention group were more likely to know the benefits of screening and to participate in CC screening post-intervention than women in the control group (90% vs. 73%) [46]. Four studies identified information availability, such as information provided via social media on CC screening, as an important facilitator for women’s CC screening uptake [33,36,48,53]. Notably three of these four studies were situated in urban settings where women’s use of social media is typically higher than that of women living in rural areas [33,36,48,53]. One study in South Sulawesi (n = 350) found that women with sufficient CC knowledge and who have good general access to information about CC screening are between 4.5 and 6 times more likely to undergo CC screening than other women [48].

Twelve studies reported on **attitudes, motivations and perceptions** of women as facilitators for CC screening and were conducted in Yogyakarta, Central and East Java, Banten and Bali [37,38,43,49,51–54,58,60–62]. These studies demonstrated that women’s positive attitude, their perceptions of both the benefits of screening and the seriousness of CC influenced their motivation and uptake of CC screening. A positive attitude towards CC screening was found to increase women’s likelihood to undergo CC screening between 2.04 and 22.33 times (OR) [42,49,53,61]. Perceived benefits of CC screening were also found to increase women’s participation in CC screening between 1.61 and 5.21 times [49,61]. However, attitudes and perceptions are also influenced by other external factors, such as trust in screening services and by observing behaviours of peers and their experiences with CC screening. The reviewed studies indicated that women whose peers undergo CC screening are up to 4.3 times (OR) more likely to access screening themselves [53]. Perceived seriousness of CC was also observed to increase women’s motivation and likelihood by 1.17 times to access screening [61].

Four studies conducted in Central Java and Yogyakarta discussed having **health insurance and a short travel distance to CC screening services** as a demand-side facilitator. Among those, three studies discussed in particular that women **having health insurance** was found to be a facilitator for accessing CC screening across Yogyakarta and Central Java [23,41,45]. Having insurance increased women’s likelihood to access screening by 9.15 times^5^^5^As Indonesia has a decentralized healthcare system not all regencies (kabupaten) provide reimbursement for the government-run CC screening even though national policies state that CC screening is intended to be covered by the National Health Insurance Scheme [79,80]. [45]. **Short travel distance** to screening services was a facilitator identified by two studies in Central Java [23,42]. In these studies, living within 8 km of a service increased the likelihood of accessing screening by almost four times [23,42].

Eleven studies conducted in the provinces of West, Central and East Java, Banten and Jakarta discussed the influence of **education and socioeconomic status** on CC screening uptake [23,38,47,50,52,55,57,58,61–63]. Women with high school and post-secondary education were up to 1.8 times more likely to access CC screening than women with lower education [47,58]. Moreover, women with an occupation or a monthly income of more than 2 million rupiah are also more likely to access CC screening. Other facilitators for CC screening uptake identified in the reviewed studies included being aged between 30 and 42 years [55,58], being married [47,58], and having a family history of CC [33].

### Supply-side facilitators

**Counselling and support** provided by screening providers, typically via pre- and post-screening or by encouraging women to access CC screening, was identified as a supply-side facilitator in six studies and was found to have a positive impact on screening uptake [22,33,37–39,47].

Six studies set in West, East and Central Java, Yogyakarta and Jakarta found that **ease of access** in of geographic convenience and availability of free CC screening services were important facilitators for women’s uptake of services [22,33,41,42,51,53]. The availability of mobile outreach or mass screening events was also identified as a facilitator in two studies in West and Central Java [22,40]. These events improved access to CC screening especially for rural-dwelling women in areas with limited health care coverage [22]. Women in three studies in Yogyakarta, Jakarta and East Java also stated that they would attend CC screening if it is available free of charge [33,41,51].

Two studies in Central Java and Yogyakarta found that the **gender of HCP** predicts women’s comfort during CC screening services. Therefore, the availability of a female HCP had an impact on women’s CC screening behaviours, as it reduced their discomfort related to modesty, embarrassment or shame during CC screening. Employing more female CC screening providers was hence concluded to be an important facilitator to CC screening acceptability and uptake [40,41].

**Health promotion and advocacy** were discussed as facilitators in two studies conducted in West Java and Riau [22,39]. Health promotion and advocacy were found to be particularly useful when the health office collaborates with the community level such as community leaders in a joint health promotion effort. It was argued that this due to the fact that the environment of women can influence their health behaviours. Consequently, community leaders can serve as important role models and advocates for CC screening [39]. HCPs are also a common source of health promotion and advocacy and hence those two facilitators were commonly linked [22,39].

One study in Semarang, Central Java evaluated the CC screening program in 13 primary health care clinics and showed how **clear supervision and support of HCPs and ensuring quality of services** at CC screening services is essential to the delivery of CC screening [44]. Hence, the study concluded that the following factors are important facilitators for the smooth delivery of quality CC screening: clear communication between midwives and clinic managers; positive attitudes, motivation and commitment among screening providers; regular supervision and provision of feedback to midwives who provide screening; ensuring the availability and comprehension of screening guidelines for midwives; and ensuring the availability of functional screening equipment.

## Barriers to CC uptake and delivery

Twenty studies identified demand-side barriers [23,33,34,36,40,42,45–48,50,54–56,58–61,63], one study identified supply-side barriers [44], while five studies discussed both supply-and demand-side barriers [31,39,41,52,57]. Seven key themes of barriers were identified in our analysis of included literature on demand-side barriers: **lack of knowledge, awareness and lack of confidence in screening outcome or quality; low-risk perception; lack of husband’s permission and general support; fear, fatalism and shame; time and transportation constraints; and low education**. Six themes of supply-side barriers related to the delivery CC screening were identified in our analysis, including: **limited access/coverage and operating hours; lack of skilled CC screening providers; lack of advocacy and health promotion; inadequate implementation and coordination; resource constraints; and lack of communication and support for HCPs.**

### Demand-side barriers

Fourteen studies set identified **women’s lack of knowledge and awareness, and lack of confidence in screening** as crucial barriers to their uptake [22,23,31,33,34,36,40,48,51,55,57–59,62]. Twelve out of 14 studies focused on women’s lack of understanding or awareness of either CC prevention and/or where CC screening is available [22,23,31,33,34,36,40,48,50,51,58,59]. Additionally, having a negative attitude towards screening with respect to outcome expectation, trust in services and perceived quality was identified as a barrier in three studies [31,57,59]. A qualitative study with men undertaken in Central Java (n = 15) identified their lack of knowledge about CC as a barrier to screening uptake [40].

Four studies conducted in East Jakarta, Central, West and East Java identified **low-risk perception** as a demand-side barrier to CC screening [40,50,51,55]. Limited knowledge about CC can contribute to low-risk perception among women because it is asymptomatic until it reaches later stages of the disease [40,55]. Misconceptions among women about being at risk for CC was identified as a barrier as women understood themselves as being outside of ‘high-risk’ groups [40,50,51].

Six studies conducted in Central and West Java, North Sumatra, Jakarta and Yogyakarta identified **lack of husband’s permission and general support** as a barrier to women’s decision to access CC screening [22,31,41,47,50,56,59]. The requirement of a husband to consent to undergo screening was identified as a barrier in three studies [22,41,50]. A related barrier to women accessing CC screening identified in two studies was the need to explain the procedure to husbands and lack of available educational material for this purpose [22,41]. Another study in West Java found that lack of support by HCPs prevents some women from attending regular or repeat screening [47].

Ten studies conducted in West and East Java, Riau, Yogyakarta and Jakarta, identified the issues of **fear, fatalism and shame** to be demand-side barriers to screening [22,33,34,39–41,47,50,51,55]. Women feared experiencing pain undergoing screening, based either on their own prior experiences or those of their peers [33]. Fear of receiving a positive result of cancer or pre-cancer was also reported as a barrier to screening in three studies [40,50,55]. Fear of a positive test result was also linked with fatalism in three studies, which related to women’s belief that if cancer was brought about by preordained destiny, it could not be altered or cured [22,41,51]. In five studies in Yogyakarta, Riau, West Java, Central and East Java, shame or embarrassment resulting from having a pelvic exam during screening, was reported as deterring women from accessing screening or returning for repeat screening [33,39–41,47]. Having a male doctor as a screening provider was also identified as a deterrent for women’s screening uptake, and was linked directly with women’s desire to avoid feelings of shame or embarrassment [40,41].

**Time and transportation constraints** were reported as barriers to access in eight studies conducted in Yogyakarta, Jakarta, Central and West Java. Excessive travel distance was identified as a barrier for women to access CC screening, particularly for women who live far from a primary health centre or have limited means of transportation [22,31,41,42,46,50–52,57]. The barriers of time constraints and excessive travel distance were also logically linked, due to the cost and time it takes to travel long distances.

**Low education and/or socioeconomic status** were identified as barriers to women accessing the adequate information about CC screening and attending CC screening in four studies in Yogyakarta, Jakarta, Banten and Central Java [45,58,59,63]. A study with 384 women in Yogyakarta and a study with 124 women in Jakarta, each identified that the cost of seeking health services is a barrier for women to access CC screening [31,41].

### Supply-side barriers

**Limited access/coverage and operating hours** were identified as a barrier in two studies in West Java and Jakarta [22,31]. An evaluation study of seven public health centres in West Java for example, showed that CC screening was only offered on limited days and specific hours. This was found to be a barrier for women intending to access regular screening as most of them live far away from healthcare centres and need to make special arrangements to undertake a trip for this purpose [22]. Moreover, limited coverage of screening services was observed to impact women’s decision to travel long distance to access them in a study in Jakarta [31].

Another supply-side barrier identified in three studies [36,39,44] was the limited training on CC screening available for HCPs leading to a **lack of skilled HCPs** who could perform CC screening. According to an evaluation in Riau province in 2018 for example, only 31 out of 956 eligible HCPs (3.14%) working at public health centres were trained in CC screening [39]. Another evaluation at a health care centre in Central Java also showed that only three midwives were trained and permitted there to conduct CC screening, which was also reflected in a low coverage of CC screening among women of age 30–50 years (5%) attending the same healthcare centre [36].

The **lack of advocacy and health promotion** in the forms of community mobilisation and counselling regarding the importance and availability of CC screening was identified as a barrier to CC screening in three studies in West and Central Java [22,44,52]. A lack of advocacy volunteers in West and Central Java who educate women about CC within their community and thereby encourage them to access CC screening was identified as an important challenge to CC screening uptake [22,52]. Moreover, a lack of educational material posted on walls at healthcare centres was seen as a factor for low uptake and awareness of screening services [52]. Insufficient use of media and targeted counselling for women was also identified as a barrier to screening uptake in Central Java [44].

**Inadequate implementation and coordination** of national CC screening efforts was identified as a supply-side barrier in two studies in Central Java and Riau [39,44]. CC screening implementation was found to be challenged in particular by a combination of miscommunication among healthcare centre staff, lack of human resources, equipment and limited supervision of HCPs in an evaluation of a health centre in Central Java [44]. In another study in Riau, it was also evaluated that operating standards for CC could not be implemented due to inadequate facilities and infrastructure. Limited funding, information and miscommunication about requirements for the CC program implementation also caused operational issues [39].

**Resource constraints** such as lack of, or broken screening equipment, and lack of funding for screening services was a barrier for delivery of CC screening services in two studies [22,39]. In a study in Riau for example, inadequate budgets did not allow the purchase of adequate screening equipment and an unreliable electricity supply forced HCPs to switch to battery-operated examination lamps. Hence, it was identified as a barrier to CC screening delivery [39].

Additionally, a **lack of communication** between screening providers, clinical managers and directors of primary health clinics on guidelines goals and targets was observed as a barrier constraining the provision of screening services in three studies in Central Java and Riau [22,39,44]. Lack of communication was found to be a major source of confusion over roles, responsibilities and inefficient cooperation among HCPs leading to poor service execution in health centres in West Java and Riau [22,39]. Lack of communication among staff members was also attributed to a lack of regular meetings of all HCPs to ensure that appropriate screening guidelines, related responsibilities and screening program targets are continuously followed as shown in a study in Central Java [44].

Finally, the same three studies identified a **lack of support** for screening staff in the form of regular feedback and supervision as supply-side barrier [22,39,44]. Limited supervision and feedback for working screening providers also negatively contributed to HCP’s motivation, understanding and commitment towards CC program targets in Central Java [44]. A need for greater support for HCPs through regular communication and affirmation of screening goals through regular meetings was also identified in a study in Riau [39]. In West Java, HCPs also pointed out the need for more support from outside the healthcare centres such as community advocacy teams to mobilize and reach more women on the community level and to encourage them to access CC screening [22].

## Discussion

This is the first scoping review to discuss demand- and supply-side facilitators and barriers to CC screening in Indonesia. However, included research predominately focused on demand-side facilitators such as husband, family or social/peer support (14 studies); information availability, knowledge and awareness (12 studies); positive attitude, motivation and perception of benefit of screening and seriousness of CC (12 studies); higher education and socioeconomic status (11 studies) and having health insurance and short travel distance to CC screening (4 studies). Limited evidence was identified for supply-side facilitators resulting in the most commonly identified facilitators including counselling and support (6 studies) and ease of access (6 studies). Similarly, most included studies also focused primarily on demand-side barriers with most evidence focusing on lack of knowledge, awareness and lack of confidence in screening outcome or quality (14 studies); fear, fatalism and shame (10 studies); time and transportation constraints (8 studies) and lack of husband’s permission and general support (6 studies). However, evidence on supply-side barriers was particularly scarce as most studies focused on lack of skilled CC screening providers (3 studies); lack of advocacy and health promotion (3 studies); resource constraints (3 studies); and lack of communication and support for HCPs (3 studies).

The imbalance in investigation of supply and demand side factors, is problematic because it can lead to interpretations of limited data that infer victim blaming – that is blaming women for their failure to access screening [64]. Despite the apparent deficiencies and challenges highlighted in the research very few studies have focused on the supply-side aspects shaping the delivery and uptake CC screening. This pattern is also apparent in the published research on this topic focused on LMICs more generally. The tendency to focus research on individual-based (demand side) factors that prevent was also noted in a recent systematic review of 2021 on barriers and facilitators to CC screening in Southeast Asia [12,65,66]. The imbalance in focus of individual behavioural factors has also be observed within other research areas as demonstrated in a recent systematic review on NCDs by Schröders et al. (2017) [3], which noted a focus on risk factors on the individual level for the management of NCDs rather than a more efficient public health approach within Indonesia [3]. We recommend that further research into supply-side factors should be undertaken to identify strategies to address the interconnectedness of both demand- and supply-side factors. Combined supply and demand side research is needed to inform improvements in the efficiency, accessibility and quality of the CC screening program, making it more responsive to the needs and preferences of women, and subsequently improving rates of screening uptake.

This review highlights multiple gaps in the existing research on the facilitators and barriers to CC screening in Indonesia. There is a clear bias in the geographical coverage of the research towards provinces on Java island, with 34 included studies being conducted across only 10 out of 34 provinces, six of which are in Java. Additionally, the studies were mainly conducted in urban or semi-urban locations with limited inclusion of the CC screening experiences of women and HCPs in rural or remote areas. This is of concern because women in rural areas face greater cumulative barriers to accessing to CC screening. Leaving out their experiences obscures inequities between women in different provinces and with different life circumstances. Most research is also focused on married women’s CC screening experiences, excluding those of unmarried women or women within same sex unions. We recommend that additional research should be conducted with greater geographical coverage, to include women living outside urban and peri-urban centres, married and unmarried women, and women from marginalised groups.

The most commonly identified facilitators and barriers on the demand- and supply-sides, were typically interconnected and sometimes interdependent. Support to access CC screening provided by husbands or women’s close social circles was found to be both the most common facilitator, and where support was lacking the most common barrier [22,23,31,35–38,40,41,45,49,50,53,55–57,59,62]. Despite husbands’ significant influence, legally as well as culturally in Indonesia, only three studies included men’s perceptions and roles (demand-side facilitator and barrier) [22,31,40]. According to national screening guidelines Indonesian husbands’ permission is required for women to participate in CC screening, however one study reported that some men struggle to support their partners due to their lack of knowledge of CC screening, despite having a supportive attitude towards CC screening [40]. There is increasing evidence within global health research that greater male involvement in women’s reproductive health can be beneficial for women’s health outcomes, yet there remains limited evidence on effective strategies for facilitating male involvement for CC screening [67]. Two recent studies in Kenya and Ghana, parallel our finding in Indonesia, that men can be supportive of CC screening for their female partners but have limited understanding of the procedure [67,68]. Future research in Indonesia should include men’s involvement in and influence over women’s access to CC screening, and encourage the implementation and evaluation of strategies for male involvement in screening. Further research into effective strategies for peer support interventions is warranted as peers have been found to be an important demand-side facilitator on screening uptake [22,23,36–38,56,57,62]. One study conducted in Central Java found that women felt more comfortable and less embarrassed if screened together with peers in community screening events, presenting an opportunity for expanding mobile outreach efforts in areas with limited healthcare coverage [40]. We recommend upscaling research on interventions that further explore and utilise the benefits of peer support in different Indonesian communities and among different groups of women.

Knowledge, awareness and access to information were common demand-side facilitators and supply-side barriers across the included studies [22,23,31,33,34,36,40,45,46,48,49,51–53,55–59,61,62]. Women who are aware of CC screening and its benefits are more likely to access CC screening whereas women who lack awareness are less likely to. Low-risk perception and education status were also relevant, as women with higher education are more aware of how to independently access reliable information on CC [23,38,40,47,50–52,55,57,58,61–63]. The significance of education, knowledge and awareness of CC and related screening services is a frequently observed demand-side barrier and facilitator across research on CC screening in LMICs including in Southeast Asia, which affirms the findings of our review [12,66]. However, it also underlines the persistent global focus on women in research as a main source for low CC screening uptake, without contextualising uptake within broader structural issues of the respective health system. We recommend more research that investigates the impact of broader social determinants of health on screening patterns in order to achieve a more comprehensive understanding of CC screening delivery and uptake in Indonesia [69,70]. This review establishes that supply-side factors can positively influence women’s CC awareness and knowledge through counselling and support by HCPs [22,33,37–39,47]. However, we also identified a lack of trained HCPs as a common supply-side barrier [39]. The importance of the availability of skilled HCPs who can counsel women on CC appropriately in order to facilitate screening uptake has been noted in other LMICs [66,71,72]. Effective and culturally appropriate training on CC screening for HCPs is essential and research on training interventions for HCPs within Indonesia is recommended.

While knowledge of CC is important, a positive attitude, motivation and perception of benefit of screening and seriousness of CC was another common demand-side facilitator, conversely lack of confidence in screening outcome or quality was a common demand-side barrier [22,23,31,33,34,36,40,48,51,55,57–59,62]. Lack of confidence can be a consequence of negative prior experiences of screening, unavailability of quality services and a lack of trust in HCPs [31,57,59,73]. Other research in Southeast Asia has identified negative experiences with HCPs as a common barrier to CC screening uptake [66,74].

The studies reviewed established that the cost of travel and services, and travel distance are crucial for women’s decision to access screening [22,41,42,50–52,57]. This is consistent with the finding that ease of access is a common supply-side facilitator, determined by limitations in geographic coverage of the ‘national’ CC screening program within Indonesia [22,42]. Other studies in low-resource settings have shown that distance to services and related costs affect women’s decisions to access screening, especially in rural areas [69,75]. Our review also found that women are more inclined to access services if they are provided free of charge through the National Health Insurance [22,33,41,51]. This could also explain why socioeconomic status for women is an important demand-side facilitator and barrier to CC screening, especially when free government-run CC screening coverage within Indonesia only covers 12% of the eligible female population in Indonesia. The cost of screening or having health insurance has also been associated with decreased screening attendance in other LMICs, which stresses its importance for the uptake of CC screening [69].

Crucial demand-side barriers observed in ten studies were fear, fatalism and shame experienced by women [22,33,34,39–41,47,50,51,55]. Women experienced shame due to genital exposure and the invasive nature of CC screening, especially if the HCP was male [40,41]. The invasive nature of CC screening and the exposure of the genitals leads to diverse sociocultural challenges for many women and presents a frequent demand-side barrier for many women in LMICs [66,73,74]. We recommend to further investigate women’s preferences with respect to CC screening to increase their comfort during screening.

Finally, the review has highlighted that Indonesia faces significant challenges in terms of the health system capacity required to achieve wider coverage of screening services, with a lack of necessary resources and equipment for screening occurring in primary health centre settings as a main supply-side barrier to the delivery of quality CC screening [22,36,39]. Health workforce limitations were also noted in three studies [36,39,44]. Hence, we recommend focused on the specific health workforce and resource needs required to improve CC screening delivery and coverage. However, even when skilled HCPs were available, a lack of communication and support for HCPs was noted as another common supply-side barrier [22,39,44]. Lack of communication between HCPs at screening services and limited supervision has shown to decrease the efficiency and quality of services, relating to a lack of confidence and trust among women in HCPs and their services (demand-side barrier) [22,39,44]. Inadequate quality assurance of CC screening also been noted in literature in other LMICs as a key supply-side barrier to delivery of CC screening [66,76,77]. Further research on how to efficiently increase constructive monitoring and supervision for HCPs, and to investigate HCP’s needs for support, is needed in order to improve quality of care with respect to CC screening.

## Limitations

This scoping review identified a lack of research on barriers and facilitators to CC screening in Indonesia. Consequently, recommendations made have to be considered with respect to the limited evidence available. Moreover, this review drew on records about the government-run CC screening program. Potential insights about screening services provided in the private sector were not included.

## Conclusion

The review has demonstrated how crucial it is for implementers and policy makers to consider the interplay of demand-side and supply-side factors that drive the delivery and uptake of CC screening services. While this review has demonstrated some significant barriers and facilitators that link demand- and supply side, there is still a great demand for further research in order to explore these factors and to translate them into tangible solutions for CC screening uptake and delivery, to increase access to CC screening via UHC and to meet nations screening targets set for 2030.

## Supplementary Material

Supplemental Material

## Paper context

CC screening uptake in LMICs incl. Indonesia is insufficient but evidence of existing research on the range of barriers and facilitators that influence the delivery and uptake of CC screening is limited. The underlying dynamics and challenges from demand and supply-sides need to be understood to undertake meaningful improvements. This review identifies facilitators and barriers to the delivery and uptake of CC screening and offers recommendations for future research needed in Indonesia and comparable settings.
