# Supplementary material for: Facilitators and barriers for the delivery and uptake of cervical cancer screening in Indonesia: a scoping review
Source: Glob Health Action. 2021 Sep 29;14(1):1979280. doi: 10.1080/16549716.2021.1979280 (PMC8491705; doi:10.1080/16549716.2021.1979280)
Supplement: Supplemental Material [file ZGHA_A_1979280_SM0996.zip › Supplementary/22.06_CC REVIEW_Supplement 2.docx]

**Supplement 2:** Review Table of Included Articles – Facilitators and Barriers of CC Screening in Indonesia 2000-2020

| **Authors**  **& Date** | **Title** | **Characteristics of research** | **Methods applied** | **Sample** | **Facilitators identified** | **Barriers identified** | **Location/**  **setting** | **Recommendations**  **if given** |
| --- | --- | --- | --- | --- | --- | --- | --- | --- |
| Susanti (2003)  (32) | An analysis on the delay of cervical cancer patients in seeking medical check-up in Dr. Cipto Mangunkusumo National Central General Hospital Jakarta | Mixed method,  Cross-sectional study,  Primary data & secondary data | Survey,  Interviews,  Observations,  Medical documents and records | 124 women (quantitative sample)  Married  (90.3%)  40-59 years (54.8%)  4 women,  1 midwife,  1 husband  (qualitative sample) | Not specified | **Demand-Side:**  Lack of knowledge,  Negative attitude,  Medical cost,  Lack of husband support,  Distance to services  **Supply-side:**  Unavailability of Pap smear | Dr. Cipto Mangunkusumo National Central General Hospital Jakarta,  [urban] | All parties should actively participate in promoting public counseling programs and CC screening through Communication, Information and Education (KIE). This undertaking would be best coordinated by Indonesian Society of Obstetrics and Gynecology (POGI), the Indonesian Association of Midwives (IBI), the Indonesia Cancer Foundation (YKI) and the National Family Planning Coordination Board (BKKBN). More research in more provinces was suggested as well to increase evidence-based knowledge in this area. |
| Kim (2012)  (23) | Influencing Women’s Actions on Cervical Cancer Screening and Treatment in Karawang District, Indonesia | Qualitative method,  Pilot study,  Primary data | Interviews,  Focus Group Discussions | 20 women,  20 husbands,  10 doctors,  18 midwives,  3 district health officials,  16 advocacy team members | **Demand-Side:**  Social Support from husbands, relatives & friends  **Supply-Side:**  Role modelling of health workers,  Encouragement by health providers;  Free services,  Convenience of services,  Community advocacy,  Television advertising | **Demand-Side:**  Lack of knowledge, information and perception,  Difficulty explaining CC prevention to husband,  Fear and fatalism, modesty concerns,  Husband’s consent,  travel distance to services  **Supply-Side:**  Lack of communication and coordination,  Limited access to services,  Broken/Lack of screening equipment,  Limited community mobilization and advocacy,  Limited days/hours of health care centers | Karawang District,  West Java  [urban] | Gaining men’s (specifically husbands’) support for screening should be prioritized,  Counselling, coaching and informational materials should be provided by health providers to aid women explaining the procedure to their husbands,  Sustained advocacy to gain support from men and women,  Increased encouragement directed at husbands to accompany their wives to screening |
| Salmah (2013)  (58) | “Faktor Dominan yang Berhubungan dengan Perilaku Pemeriksaan Pap Smear Pada Wanita Usia Subur” [Dominant Factors Associated with Pap Smear Examination Behavior in Women of Fertile Age] | Quantitative method,  Cross-sectional study,  Primary data | Observation | 50 women  (25-45 years) | **Demand-Side:**  Social support,  High knowledge,  High education,  High income | **Demand-Side:**  Negative attitude;  Increased travel distance to CC screening services | Bekasi Regency,  West Java  [urban] | Not Specified |
| Wahyuni (2013)  (36) | “Faktor-Faktor yang memepengaru Hi Perilaku deteksi dini Kanker Serviks di Kecamatan Ngampel Kabupaten Kendal Jawa Tengah” [Factors Affecting Behaviour of early detection of cervical cancer in sub-district Ngampel Kendal District, Central Java] | Quantitative method,  Non-experimental with correlation study,  Primary data | Questionnaire | 80 women | **Demand-Side:**  Husband Support,  Women 3 times more likely to seek VIA exam if husband is supportive | Not Specified | Kendal Regency,  Central Java  [rural] | Explore other external factors that alter women’s VIA seeking behavior and the significance of husband role in health promotion efforts |
| Dewi (2014)  (57) | “Faktor-Faktor yang Berhubungan dengan perilaku pencegahan kanker serviks pada wanita usia Subur”[Factors related to cervical cancer prevention behavior in women of fertile age] | Quantitative method,  Analytical survey research,  Primary data | Questionnaire | 99 women, (married,  25-35 years) | **Demand-Side:**  Women with good social support have greater CC prevention-health behavior (44 women/86% of total) or are 10 times more likely to use CC screening,  Women at risk (categorized as <20 to >35 years) have greater knowledge about CC than women not at risk | **Demand-Side:**  Women with poor social support are less likely to have good CC prevention behaviours or access CC screening.  [Only 40% of these women would have good CC prevention behaviours such as being married, being monogamous, non-smoking, balanced nutrition intake, regular exercise and attending secondary CC prevention] | 6 Rukun Warga (Villages),  Rejosari village,  Central Java  [urban] | Not specified |
| Anggraeni (2016)  (44) | Path Analysis and Theory of Planned Behavior on Using PAP SMEAR as early detection of cervical cancer in Sewon I Community Health Center, Yoyakarta, Indonesia | Quantitative method,  Observational analytic study, cross sectional design,  Primary data | Questionnaire | 96 women,  Married (85%),  aged >35 years | **Demand-Side:**  Attitude,  Behaviour,  Subjective norms [Indirect]  Intention & Perception of Control Behaviour  [Direct]  [Theory of Planned Behaviour] | Not specified | Bantul,  Yogyakarta  [urban] | Further research to focus on direct relationship and indirect behaviour for Pap smear uptake based on planned behaviour by adding a variable for the formation of attitudes |
| Parapat (2016)  (37) | “Factors related to behaviour for early detection of cervical cancer with visual inspection method with acetic acid in Puskesmas Candiroto, Temanggung District”  [Faktor Faktor yang Berhubungan dengan Perilaku Deteksi Dini Kanker Leher Rahim Metode Inspeksi Visual Asam Asetat di Puskesmas Candiroto Kabupaten Temanggung] | Quantitative method,  Cross-sectional study,  Primary data | Questionnaire | 100 women,  Married,  30-50 years | **Demand-Side:**  Information availability,  Husband’s support and permission,  Friend support and being invited by close friend (who they trust) to be screened together | **Demand-Side:**  Did not know about availability of CC screening at study puskesmas  **Supply-Side:**  Not all midwives receive training and permission to conduct VIA exam (only 1 doctor and 3 midwives at puskesmas are trained) | Temanggung,  Central Java  [rural] | Not Specified |
| Tarigan (2016)  (60) | “Factors Related to WUS (Wanita Usia Subur) Behavior in Early Detection of Cervical Cancer in Dwikora Village, Medan, 2014”  [Faktor-Faktor Yang Berhubungan Dengan Perilaku WUS Dalam Deteksi Dini Kanker Serviks di Kelurahan Dwikora Medan Tahun 2014] | Quantitative method,  Cross-sectional study,  Primary data | Survey | 110 women,  30-50 years | Not specified | **Demand-Side:**  Lack of knowledge  Negative attitude towards CC screening  Lack of family/husband support,  Low education,  Low socioeconomic status/no occupation | Dwikora Village,  Medan City,  North Sumatra  [urban] | More health promotion efforts to raise awareness and knowledge about CC and screening across different sectors |
| Afsah (2017)  (42) | Perceived Barriers of Cervical Cancer Screening Among  Married Women in Minggir, Godean, Gamping Sub-Districts,  Sleman District Yogyakarta | Quantitative method,  Cross-sectional study,  Primary data | Questionnaire  [Champion Health Beliefs Model) | 384 women,  Married,  Muslim,  30-55 years | **Demand-Side:**  Health insurance  **Supply-Side:**  Services free of charge,  Employ more female doctors | **Demand-Side:**  Embarrassment,  Shame,  Exam painful and time-consuming,  Fatalism,  Needing to explain procedure to husband and receive permission  Perceived barrier scale (the higher the less likely women will seek screening services)  Male doctor,  Cost,  Distance to services | Sleman District,  Yogyakarta  [urban & peri-urban] | Health Promotion to address perceived barriers in order to increase CC screening uptake |
| Wakhidah (2017)  (38) | The influence of personal factor, husband’s support, health workers and peers toward the use of IVA screening among women of reproductive age in the regency of Karanganyar | Quantitative method,  Analytic observational study,  Cross-sectional design,  Primary data | Questionnaire | 150 women  (50 cases, 100 controls) | **Demand-Side:**  High perceived threats,  High perceived high benefits,  High perceived obstacles,  High Self-efficacy [Direct]  High perceived susceptibility,  High perceived seriousness,  High husband’s support,  High peer support  [Indirect]  **Supply-side:**  High health workers support  [Indirect] | Not specified | Regency of Karanganyar,  Central Java  [rural] | Not specified |
| Saraswati (2017)  (45) | Analysis of the Implementation of the early detection services for cervical cancer with visual inspection method with acetic acid (IVA) in Puskesmas/ Public Health center in Semarang city [Analisis Implementasi Program Deteksi Dini Kanker Serviks Melalui Metode Inspeksi Visual Asam Asetat (IVA) Di Puskesmas Kota Semarang] | Qualitative method,  Primary data | Interviews,  Descriptive observational method,  Program evaluation | 5 midwives,  1 head of Infectious Disease Semarang District Health Office,  5 head of puskesmas,  5 women,  30-45 years | **Supply-Side:**  Regular clear communication,  great attitude, motivation and commitment of health care staff,  regular supervision of midwives with feedback,  availability and explanation of program targets and guidelines to midwives,  sufficient equipment | **Supply-side:**  Lack of communication between midwives and head of puskesmas,  Lack of coordination and leadership,  Lack of support for midwives,  No clear communicated program targets or guidelines,  No distribution of guidelines to midwives,  Midwives unaware of program targets,  Fragmented organizational structure,  Health cadres don’t have clear guidelines on their duties, function or responsibility  Limited supervision and feedback for screening staff,  Lack of regular feedback and meetings,  Lack of outreach and health promotion events;  Lack of counseling and outreach as many women are unaware of CC screening | 13 puskesmas,  Semarang City,  Central Java  [urban] | Need to strengthen coordination, monitoring and evaluation of CC screening program,  Need to organize regular meetings across sectors with midwives, head of puskesmas and other staff to enable monitoring and evaluation,  More counseling and outreach events needed. |
| Wardhani (2017)  (39) | Path analysis on the determinants of Pap smear utilization for cervical cancer early detection in women of reproductive age | Quantitative method,  Analytic observational study,  Cross-sectional design,  Primary data | Questionnaire | 200 women | **Demand-Side:**  Good attitude,  Higher education,  Good family support,  good perception of service quality,  Good peer support  [Indirect]  Easy and convenient access to health service e.g. short distance [Indirect]  **Supply-Side:**  Good health personnel support [Direct] | Not specified | Cilongok and Ajibarang sub-districts,  Banyumas,  Central Java  [rural] | Not specified |
| Wijayanti (2017)  (61) | Factors Influencing Women in Pap Smear Uptake | Quantitative method,  Primary data | Questionnaire | 86 women,  <25 >55 years | **Demand-Side:**  Women with low perceived barriers are 3.52 times more likely to receive pap smear | **Demand-Side:**  Women with high perceived barriers are less likely to receive a pap smear | Badung Regency,  Bali  [urban] | More research needed in social and cultural barriers to pap smear uptake |
| Anwar (2018)  (24) | Determinants of cancer screening awareness and participation among Indonesian women | Quantitative method,  Indonesian Family Life Survey,  Cross-sectional study,  Secondary data | Survey,  Questionnaire | 5397 women, aged 40 and older,  Not married:(1423 women/26%),  Married: (3974 women/74%) | **Demand-Side:**  Participating in social activities,  Higher education and household expenditure,  Marital status,  Urban residence,  Having health insurance  **Supply-side:**  Access in terms of  Short distance to healthcare provider (<10 min) | **Demand-Side:**  Married women are more aware of CC screening than unmarried women | 13 out 27 provinces that existed at the same [not specified] | It is advised that access to healthcare and social participation could improve CC service uptake for socioeconomic disadvantaged women |
| Nordianti (2018)  (46) | “Determinan Kunjungan Inspeksi Visual Asam Asetat di Puskesmas kota Semarang”  [Determinants of Acetic Acid Visual Inspection Visits at Puskesmas Semarang City]   \|  \| \| --- \| | Quantitative method,  Observational cross-sectional research,  Primary and secondary data | Questionnaire,  Medical records | 44 women, married,  16-49 years | **Demand-Side:**  Women with good family support (80%) are more likely to seek early CC screening,  74% of women with health insurance are likely to receive a VIA examination (9.15 times more likely to do VIA exam),  Women with known CC risk factors are more likely to receive a VIA test | **Demand-Side:**  More women with low education will take a VIA examination  (46% of participating women) | Catchment area of 13 puskesmas,  Semarang City,  Central Java  [urban] | Most women in the study expressed positive attitudes towards early CC detection but did not receive CC screening. Therefore, other associations have to be closer examined in order to increase CC screening uptake. |
| Suhaimi (2018)  (40) | Implementation of the Cervical Cancer Early Detection Program (Ca Cervix) with Visual Inspection with Acetic Acid Method (VIA) in Kampar Regency | Qualitative study with narrative research,  Primary data | Interviews | 12 informants,  1 head of P2P Division of Kampar Health Office, 5 Puskesmas Head, 2 P2 Program Managers of puskesmas, 3 midwives | **Supply-side:**  Health promotion activities and counseling about CC screening has improved women’s uptake of VIA exam;  Cross-sectoral cooperation for health promotion (government and private e.g. community leaders) found to be effective for widespread public health efforts/promotion. | **Demand-Side:**  Embarrassment, shame and reluctance to receive CC screening,  Transportation cost to health center with CC screening;  **Supply-Side:**  Operating Procedure Standards for CC screening exist but are poorly implemented due to inadequate facilities and infrastructure;  Lack of trained midwives (only 31/956 midwives are trained in VIA examination or 3.24%), only 1 trained midwife per puskesmas in catchment area,  Lack of funding to provide equipment and tools for CC screening; need to use battery-operated examination lamps as electricity not guaranteed,  inefficient logistics;  miscommunication and lack of information for program implementers causes operational issues. | Kampar District,  Puskesmas  Riau  [rural] | Strengthen advocacy and cross-sectoral cooperation with all stakeholders to increase coverage and reach of health promotion efforts,  Dissemination of information about CC through print and social media,  Strengthen training for health providers,  Strengthen implementation of operating standards for CC screening,  Lack of specific allocation of funds for program development through APBN and APBD funds,  Collaboration with community and religious leaders needed,  Need to target experienced shame and fear of women |
| Saptowati (2018)  (62) | Biopsychosocial Determinants of Visual Inspection Acetic-Acid Test Uptake in Sragen, Central Java | Quantitative method,  Analytic observational study,  Cross-sectional design,  Primary data | Medical records,  Questionnaire | 200 women | **Demand-Side:**  Stronger Intention,  Perceived benefit,  High parity  [Direct]  Positive attitude;  Stronger subjective norm,  High self-efficacy,  Good knowledge,  Higher education,  Stronger perceived susceptibility,  Stronger perceived seriousness [Indirect] | **Demand-Side:**  High perceived barrier  [Direct] | Sragen,  Central Java  [urban] | Not specified |
| Silalahi (2018)  (47) | Effectiveness of audiovisual and booklet as Education Media to  Improving IVA Screening behavior | Quantitative method,  Quasi-experimental Non-Equivalent Case-Control Design,  Primary data | Survey (Pre-Post Intervention) | 79 women (Intervention/  control group),  30-50 years | **Demand-Side:**  Increased knowledge of CC,  Women more likely to undergo VIA after short health education provided compared to women without short health education provided (90% vs. 73%) | **Demand-Side:**  Health education alone had no impact on perceived barriers to CC screening | Pacarkeling Puskesmas [Pacarkeling and Pacarkembang Village],  Surabaya,  East Java  [urban] | Health education should be provided and expanded through health cadres, more research needed to evaluate perceived barriers and how to address them to improve CC screening |
| Wulandari (2018)  (48) | “Faktor-faktor yang Berhubungan dengan Perilaku Pemeriksaan Inspeksi Visual Asam Asetat (IVA) pada Wanita Usia Subur (WUS) di Puskesmas Sukmajaya Tahun 2016” [Factors Related to Behaviour of Visual Inspection of Acetic Acid (IVA) of Childbearing Women in Puskesmas Sukmajaya 2016] | Quantitative method,  Analytical observational study,  cross-sectional design,  Primary data | Questionnaire | 146 women;  Married (93%),  Not married (7%),  Over 40 years (73%),  Housewives (62%),  High school graduates (44%),  Low income or husband with low income (93/95%) | **Supply-Side:**  VIA related information obtained through health worker or cadre,  Support from health worker or cadres  **Demand-Side:**  Women with good income, education (high school and higher) & being married  are likely to take VIA exam. | **Demand-Side:**  Fear,  Shame  **Supply-Side:**  Lack of support  by health workers/cadres | Puskesmas Sukmajaya,  Depok City,  West Java  [urban] | It is recommended to improve available services; implement pre-screening counseling and include men in health promotion efforts. |
| Armadhani (2019)  (63) | Path Analysis of Pap Smear Uptake in Women of Reproductive Age in Tegal, Central Java | Quantitative method,  Analytic observational study,  case-control design,  Primary data | Questionnaire | 200 women | **Demand-Side:**  Stronger Perceived threat of CC,  Stronger Perceived Benefit,  higher self-efficacy [Direct]  Perceived susceptibility/  Seriousness,  Cues to action, education, knowledge and peer support [Indirect] | Not Specified | Tegal,  Central Java  [urban] | Not Specified |
| Kholifah (2019)  (43) | Factors Affecting the Use of Visual Inspection Acetic Acid Test: Multilevel Analysis on Contextual Effect of Health Center | Quantitative method,  Analytic observational study,  Cross sectional design,  Primary data | Questionnaire | 225 women,  20-49 years | **Demand-Side:**  Good Intention,  Positive Attitude,  Positive outcome expectation, high imitation of behavior,  type of contraceptive method,  high self-efficacy,  time to attend health center,  Short distance to health services (<8km)  **Supply-Side:**  Public health Center accreditation | **Supply-side:**  Limited access in terms of long distance to health service | Solo and Karanganyar,  Central Java  [rural and urban] | Not Specified |
| Nuryana (2019)  (49) | The relationship of knowledge and Information Availability on Cervical Cancer early Detection in Childbearing Age Couples with VIA Method in Takalar | Quantitative method,  Observational cross-sectional design,  Primary and secondary data | Questionnaire,  Medical records | 350 women,  20-45 years | **Demand-Side:**  Women with sufficient CC knowledge 4,5 times more likely to participate in early CC detection with VIA method;  Women with more information availability on CC detection are 5.9 times more likely to participate in VIA exam | **Demand-Side:**  Limited knowledge and information availability | North Galesong Community Health Center,  Takalar Regency,  Makassar City,  South Sulawesi [urban] | Health workers are recommended to provide health education and counseling for early CC detection and screening which also involves husbands and families of women; health information needs to be made accessible for women across their lifespan as different generations access health information differently. |
| Spagnoletti (2019)  (41) | A Qualitative Study of Parental Knowledge and Perceptions of Human Papillomavirus and Cervical Cancer Prevention in Rural Central Java, Indonesia: Understanding Community Readiness for Prevention Interventions | Qualitative method,  Primary data | Focus Group Discussions,  Interviews | 57 participants (39 women:  28-40 years,  15 men:  35-45 years) | **Demand-Side:**  Women: Knowledge about early detection of CC; having peers screened as well during community mass screening programs (peer/social support)  **Supply-Side:**  Mobile outreach,  Employ more female doctor | **Demand-Side:**  Women: Limited knowledge about CC, Male doctor,  Embarrassment during VIA exam,  not knowing where to get tested, cost, fearing a positive result, not understanding risks  Men: not heard or limited understanding about screening and CC symptoms,  being asymptomatic and not perceiving need for testing | Gebang, Sekartejo, Winong Kidul & Gintungan villages,  Central Java  [rural] | Need to address women’s concerns of embarrassment with male doctors to increase screening uptake,  Additional education for health workers needed;  Tailored education materials need to be developed for women, adolescents and men,  Strengthen CC related health education for women and men and address women’s fears and concerns. |
| Sunarta (2019)  (50) | Path analysis  on the determinants  of visual inspection acetic acid utilization on early detection of cervical cancer: application of health belief model theory | Quantitative method,  Analytic observational study,  case-control approach,  primary data | Questionnaire | 200 women (120 women never screened,  50 women screened) | **Demand-side:**  High perceived vulnerability, high perceived severity,  high perceived benefit, high cues to action, high self-efficacy, good attitude; good husband support  [Direct]  Good Knowledge,  Easy access to healthcare,  (convenience, travel distance  etc.) [Indirect] | Not specified | Wongsorejo Public Health Center,  Banyuwangi,  East Java  [urban] | Not specified |
| Mulyati (2019)  (51) | Association of perceived barriers factor and participation of women in early detection of cervical cancer | Quantitative method,  Descriptive cross-sectional study,  primary data | Questionnaire | 190 women,  Married,  70% >30 years | **Demand-Side:**  Women >50yo have reduced odds of having fear of positive result;  Women in their 30s are more likely than women in their 20s to participate in CC screening,  Higher education level, parity and income. | **Demand-Side:**  Misconceptions like CC screening only for women at “high-risk”,  screening will be expensive.  Fear of positive result, needing husband’s permission, having no time for screening; long travel distance to CC screening services | Public Health Center,  Bandung City,  West Java  [urban] | Development of health information and promotion need to take into consideration the perceived barriers to improve CC screening uptake |
| Wantini (2019)  (35) | “Deteksi Dini Kanker Serviks dengan Inspeksi Visual  Asam Asetat (IVA)” [Early Detection of Cervical Cancer by Visual Inspection  Acetic Acid (IVA)] | Quantitative method,  Analytical survey research,  Cross-sectional design,  Primary data | Questionnaires,  Interviews | 350 women,  19-49 years | **Demand-Side:**  Knowledge about benefit of CC screening | **Demand-Side:**  Lack of Knowledge about CC screening,  Fear,  No time (31/8.9%),  No complaints or symptoms  (24/6.9%) | Kalasan Public Health Center, Kalasan Sub-District,  Sleman Regency,  Yogyakarta  [peri-urban] | This research should inform CC screening information material for women provided through health providers,  Apply other sampling methods in future research to cover catchment area of all public health centers within the area,  Factors such as fear, lack of knowledge about CC screening and experiencing no symptoms influence women’s decisions to attend CC screening and need to be further investigated |
| Winarto (2019)  (52) | Cervical Cancer Related Knowledge, Attitude and Behaviour Among Women in Makasar District Primary Health Care Centre in 2018 | Qualitative method,  Descriptive cross-sectional study,  Primary data | Interviews | 105 women,  married,  Mean 30 years | **Demand-Side:**  Knowledge,  Positive Attitude  **Supply-side:**  Free services | **Demand-Side:**  Distance from home to health center,  Low-risk perceptions of cervical cancer,  poor knowledge about available screening services (60%); fear,  Lack of symptoms and fatalism. | Makasar District Primary Health Care Center,  East Jakarta  [urban] | More health promotion efforts needed especially at primary health care level as women are unaware of CC screening services. |
| Wulan (2019)  (53) | The Effect of Community Health Center on Visual Acetic Acid Uptake in Pati, Central Java | Quantitative method,  Observation study, cross sectional design,  Primary data | Questionnaire | 232 women; | **Demand-Side:**  High perceived susceptibility & seriousness, threat, barrier, benefit & economic status | **Demand-Side:**  Low cues to action, self-efficacy, social capital,  travel distance  **Supply-Side:**  Lack of information, lack of counseling, uncomfortable screening rooms | 29 Community health centers,  Pati  Central Java  [urban] | Not specified |
| Martaningrum (2020) (54) | Contextual Effect of Community Health Center on Visual Inspection Acetic Acid Uptake in Magelang, Central Java: A Multilevel Analysis | Quantitative method,  Observational study,  Cross-sectional design,  Primary data | Questionnaire | 200 women,  30-50 years | **Demand-Side:**  Good knowledge,  Observational learning,  Strong behavioural reinforcement,  Strong cues to action, good access to information, strong family support,  Positive attitude,  Strong self-efficacy,  Strong outcome expectation  **Supply-Side:**  Quality and accessibility of public health center | Not specified | 25 Community health centers, Magelang,  Central Java  [urban] | Not specified |
| Muhtih (2020)  (55) | Internal Locus of Control as a Driving Factor of Early Detection Behavior of Cervical Cancer by Inspection Visual of Acetic Acid Method | Quantitative method,  Cross-sectional design,  Primary data | Questionnaire | 393 women,  71% housewives,  20-50 years | **Demand-Side:**  High locus of control | **Demand-Side:**  Low locus of control | 9 Community  health centers,  Kedirir City  East Java  [urban] | Not specified |
| Saputra (2020)  (59) | Working Women Behavior on Cervical Cancer as Participants of National Health Insurance | Quantitative method,  Correlation analysis,  Primary data | Questionnaire | 158 married women,  employed with Serang City Health Services | **Demand-Side:**  Good knowledge,  High self-motivation,  Higher education,  Higher parity (>2 children),  Length of marriage (11-20 years),  Age (30-42 years) | **Demand-Side:**  Less knowledge,  Low self-motivation,  Low education,  Older age  (43-55 years) | Serang City Health Services,  Serang City  Banten  [urban] | Not specified |
| Arimurti (2020)  (64) | “Relationship of Education with early detection Behaviour for Cervical cancer in Women in Kebon Kapala Bogor” [Hubungan Pendidikan Dengan Perilaku Deteksi Dini Kanker Serviks Pada Wanita Di Kelurahan Kebon Kalapa Bogor] | Quantitative method,  Cross-sectional design,  Secondary data | Survey | 1,226 women | **Demand-Side:**  Good education (1,8 x more likely to undergo cervical cancer screening) | **Demand-Side:**  Low education | Ministry of Health,  Kelurahan Kebon Kalapa,  Jakarta  [urban] | Further qualitative research needed to evaluate other variables and their correlation with Cervical Cancer screening. |
| Umami (2020)  (34) | Knowledge, Barriers, and Motivation Related to Breast and Cervical Cancer Screening Among Women in Bojonegoro, East Java: A Qualitative Study | Qualitative Method,  Primary data | Focus Group Discussions, Interviews | 20 women,  20-65 years,  married (65.2%) | **Supply-Side:**  Information provided through health facilities,  Free services  **Demand-Side:**  Family history of CC,  Information provided through social media | **Demand-Side:**  Fear of examination,  Embarrassment,  Peer stories reporting pain during examination,  Concerns over modesty,  Awareness of screening services; Not experiencing signs or symptoms. | Community setting,  Ledok Kulon village, Bojonegoro Regency,  East Java,  [peri-urban] | Access to services alone is not sufficient and needs to be complemented with community mobilization |
| Widayanti (2020)  (56) | Mother’s knowledge and attitudes towards Visual Acetate Acid Inspection test in Surabaya | Quantitative method,  Analytical cross-sectional study,  Primary data | Medical records | 126 women  20-40 years | **Demand-Side:**  Family support, especially from husband,  Education makes women more likely to obtain the right information and the more knowledgable they are about CC,  Income more than Rp >2,000,000/  Month, occupation, marital status, age | **Demand-Side:**  Only seek services when feeling symptoms,  Lack of awareness and knowledge about CC and screening, fear of positive VIA exam | Family section of the Maternal and Child Health (MCH) Center,  Surabaya,  East Java,  [urban] | Not specified |
