# Supplementary figures and images for: Facilitators and barriers for the delivery and uptake of cervical cancer screening in Indonesia: a scoping review
Source: Glob Health Action. 2021 Sep 29;14(1):1979280. doi: 10.1080/16549716.2021.1979280 (PMC8491705; doi:10.1080/16549716.2021.1979280)

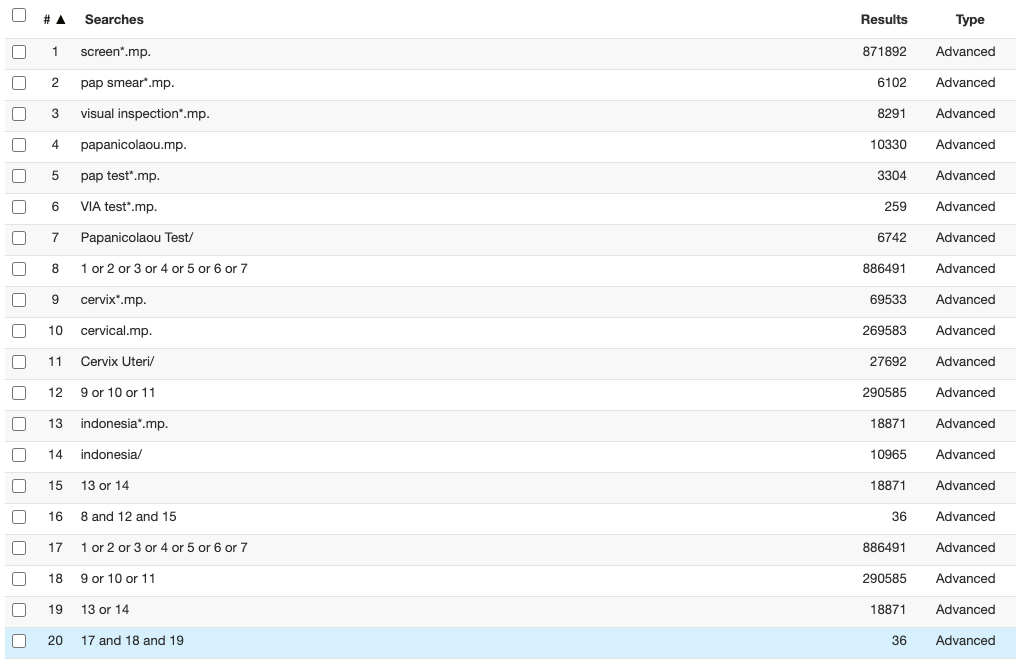

Supplement: Supplemental Material [file ZGHA_A_1979280_SM0996.zip › Supplementary/16.03_GR_Supplement 1_Medline Ovid Search.docx]

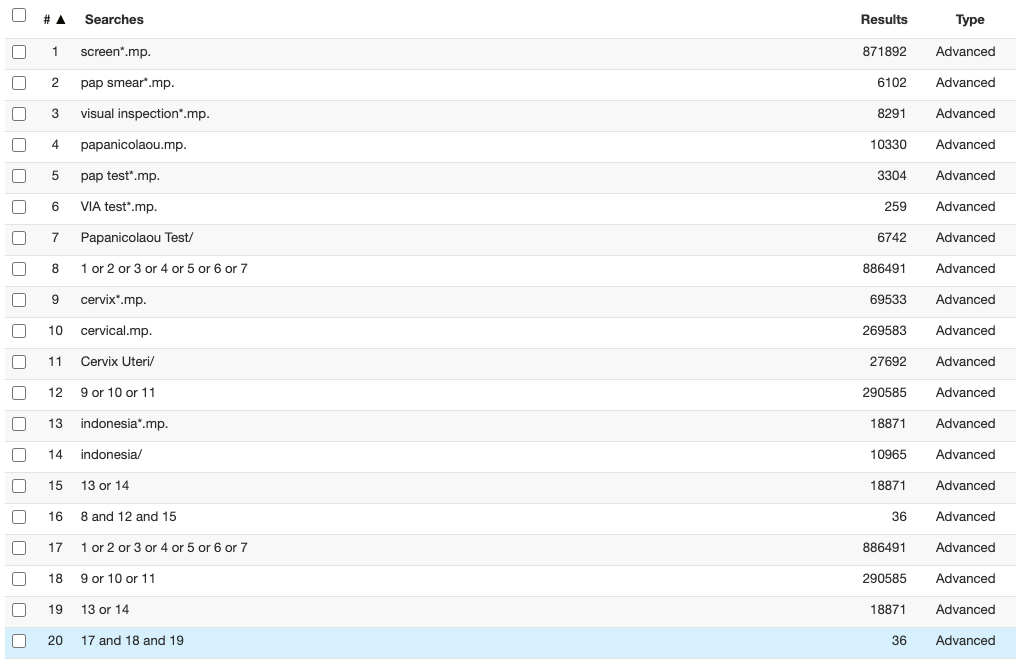

Supplement: Supplemental Material [file ZGHA_A_1979280_SM0996.zip › Supplementary/22.06_GR_Supplement 1_Medline Ovid Search.docx]
